# A Rare Case of a Giant Intramuscular Lipoma of the Upper Extremity in a Pediatric Patient

**DOI:** 10.7759/cureus.53575

**Published:** 2024-02-04

**Authors:** Jeffrey Ai, Rakel Zarb, Sarah Cassidy, Kant Lin

**Affiliations:** 1 Plastic Surgery, Medical College of Wisconsin, Milwaukee, USA

**Keywords:** intramuscular lipoma, soft tissue neoplasms, lipoma, humans, child

## Abstract

Although lipomas are the most common benign soft tissue tumors, the non-infiltrating intramuscular subtype is relatively uncommon. As these masses typically present between the ages of 40 and 70, few cases have been reported in the pediatric population. We present a case of a giant intramuscular lipoma of the biceps brachii in an adolescent. He presented with a slow-growing, tender mass and had no neurovascular compromise of the limb. MRI was utilized to visualize the mass, and a muscle-sparing excisional biopsy was performed. Histologic evaluation confirmed a diagnosis of a benign lipoma. The patient went on to heal without a functional deficit. Large, growing soft tissue masses warrant work-up to rule out malignancy. Advanced imaging and excisional biopsy are necessary to confirm the diagnosis of a benign giant intramuscular lipoma, which is especially rare within the pediatric population. We discuss the prevalence and treatment of intramuscular lipomas, including a literature review of reports in the pediatric population.

## Introduction

Lipomas are the most common soft tissue tumors, occurring in two percent of the population [1]. Intramuscular lipomas are an uncommon subtype with an incidence of less than one percent of all lipomas, with the majority occurring between the ages of 40 and 70 years [2]. The definition of giant lipomas varies in the literature, including a minimum dimension of 5 or 10 cm or a minimum weight of 1000 grams [3,4]. In the pediatric population, the occurrence of intramuscular lipomas is rare, and there have been no reported cases in the adolescent population. In this report, we present a case of a giant intramuscular lipoma of the biceps brachii of an adolescent male, as well as a review of the current literature.

## Case presentation

**Figure 1:**
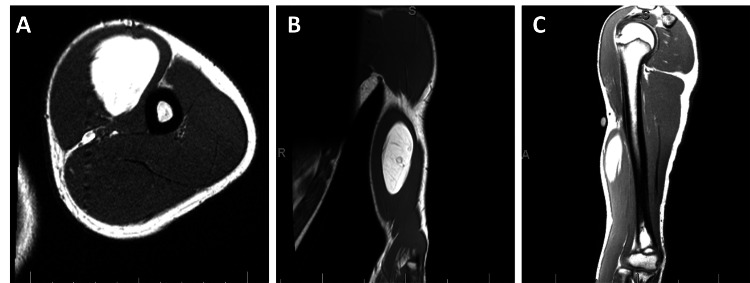
Transverse (A), coronal (B), and sagittal (C) plane T1-weighted MRI of the patient's upper extremity prior to excision.

Informed consent was obtained from the participant and his guardian. The patient is a 14-year-old otherwise healthy male who presented to the clinic for the evaluation of a soft tissue mass of the left upper extremity. The mass had been slowly growing over the course of two years. He reported occasional focal tenderness with palpation. On physical examination, there was a large palpable mass of the anterior brachium. His exam was reassuring for an absence of compression, including intact pulses with no motor or sensory deficits. Magnetic resonance imaging (MRI) was obtained, which demonstrated a well-encapsulated lipomatous mass measuring 9 by 3.5 by 4 cm within the biceps brachii muscle without concerning features (Figure 1).

**Figure 2:**
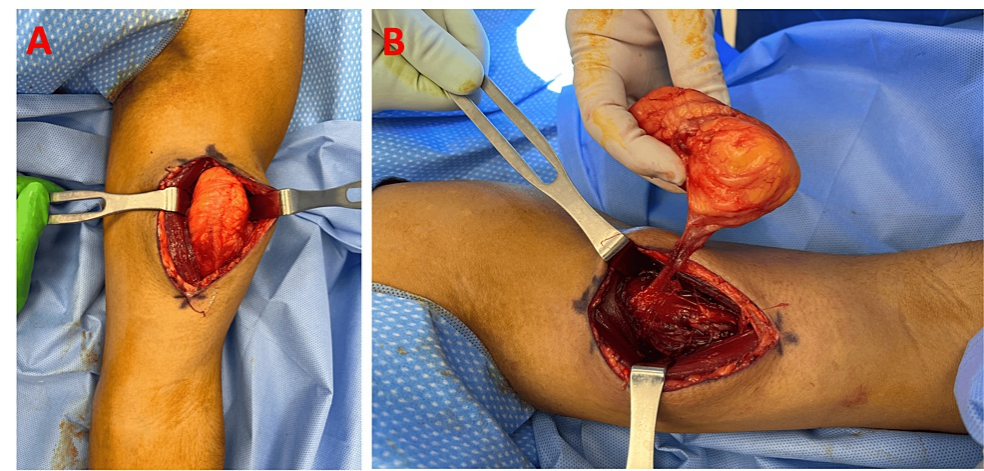
(A) Intraoperative photo of lipoma in situ of patient's bicep with retractors assisting in visualization. (B) Lipoma immediately prior to complete excision.

**Figure 3:**
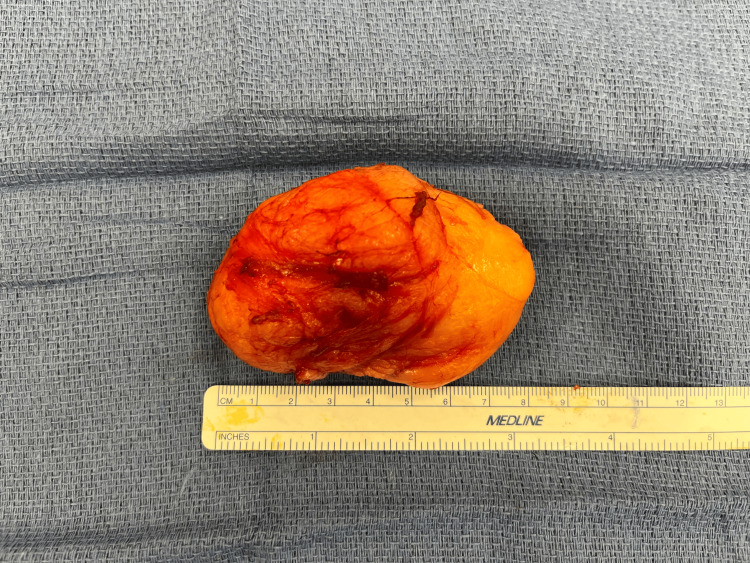
Intramuscular lipoma following wide excision with a ruler to scale for dimensions.

**Figure 4:**
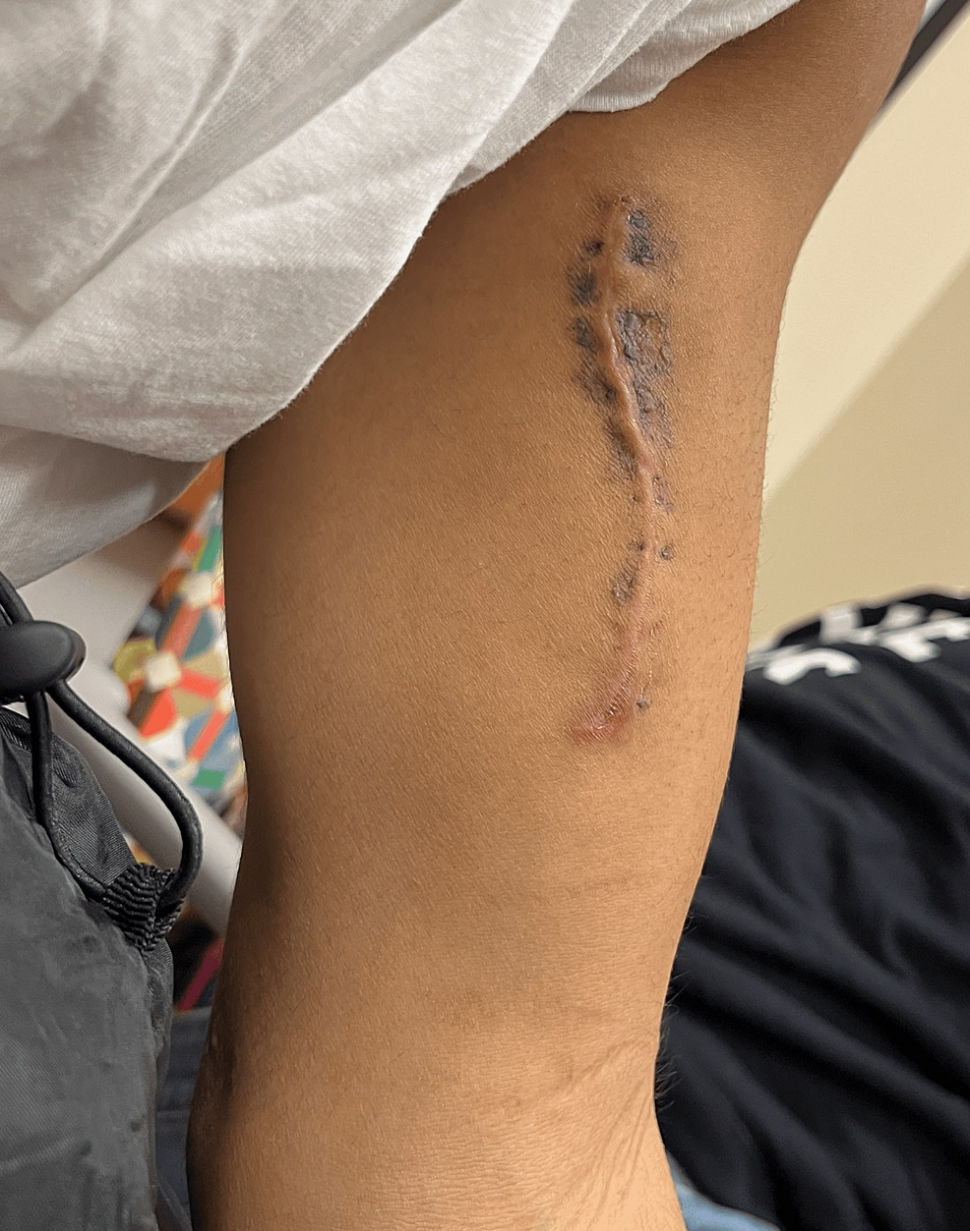
Patient's arm four weeks postop demonstrating no signs of recurrence and a well healing scar.

Given the size, pain, and history of growth, we proceeded with excisional biopsy in the operating room. A longitudinal “lazy S” incision was designed over the biceps brachii. The muscle fibers were longitudinally separated to avoid transection and therefore preserve function and minimize post-operative pain. A well-encapsulated lipomatous mass measuring 9 by 4.5 by 6 cm was encountered between the short and long heads of the biceps brachii. The mass was circumferentially dissected and removed en bloc (Figures 2, 3). The surgical site was closed over a drain and a compressive dressing was placed. Histologic evaluation supported the diagnosis of benign lipoma (Figure 4). The patient was discharged the day following the surgery with instructions to keep the arm immobilized for two weeks. Drain was removed one week postop, and a follow-up examination four weeks postop showed no evidence of recurrence with a well-healing scar.

## Discussion

Lipomas are benign soft tissue mesenchymal neoplasms derived from mature adipocytes. They can be classified based on depth into subclasses of dermal, subcutaneous, and subfascial lipomas. Subfascial lipomas can be further categorized as intermuscular and intramuscular. Intermuscular lipomas grow between large bundles of muscle fibers, whereas intramuscular originate between muscle fibers within bundles. Intramuscular lipomas consist of less than 1% of all lipomas and only 1.8% of all primary adipose tumors [2]. The majority of intramuscular lipomas occur between the ages of 40-70 [2]. The soft tissue mass in this case was a well-defined intramuscular lipoma, an exceedingly rare finding in the pediatric population.

To analyze the epidemiologic features of intramuscular lipomas in the pediatric population, we searched literature published in the English language on PubMed and reviewed all available reports (Table 1).

**Table 1:** Literature review describing characteristics and treatment of pediatric intramuscular lipomas with “-“ indicating information not found within the report.

Author	Patient Age/Sex	Anatomic Site	Max. Diameter (cm)	Imaging Modality	Presenting Symptoms	Treatment	Recurrence
Adbelmohsen et al., 2019 [5]	4 yr/M	Small Intestine	4	Ultrasound	Abdominal pain and non-bilious vomiting	Resection	None
Lee et al., 2004 [6]	7 yr/ -	Hand	7	MRI	-	Resection	-
Shiraki et al., 2002 [7]	8 yr/F	External Ocular Muscle	-	MRI	Elevated Mass	Resection	-
Vincent et al., 2017 [8]	3 mo/M	Glabella	1.5	MRI	Elevated Mass	Resection	None
Bao et al., 2005 [9]	8 yr/F	Lower Extremity	9.3 and 12	Unknown	Palpable Mass	Resection	Yes
Pierron et al., 2009 [10]	5 yr/M	Buttock	6.5	-	-	Resection	-
Kendrick et al., 2022 [11]	4 yr/M	Gastrocnemius	-	MRI	Difficulty ambulating, intermittent pain	Resection	-
Gondowardojo et al., 2017 [12]	3 yr/M	Back	14.1	CT	Elevated mass	Resection	-

Since the earliest report in 2002, only eight pediatric intramuscular lipoma cases have been published. Of cases with available gender information, 5/7 (71%) occurred in males, similar to the reported number of 62% in adults [13]. Three occurred in the extremities, two in the trunk, two in the head, and one in the viscera. Of reported cases, ours has the oldest pediatric patient at 14 years with mass growth at age 12.

Classically, intramuscular lipomas present as slow-growing and painless masses that are soft and mobile. Functional deficits or pain may arise as the tumor grows and produces a mass effect. Indications for radiologic imaging prior to surgery of lipomatous lesions may include large size (> 5 cm), rapid growth, pain, fixation to surrounding tissues, and subfascial location that require imaging to rule out malignancy [14]. Due to these considerations, intramuscular lipomas typically require advanced imaging to establish a diagnosis. Depending on the anatomic location, imaging may also be useful for pre-operative planning.

Computerized tomography (CT), ultrasound, and MRI are options for further characterizing masses. MRI, the preferred modality for soft tissue visualization, provides powerful anatomic details for distinguishing between lipomatous masses. The mature adipose tissue in intramuscular lipomas shows strikingly high signal intensity on both T1 and T2 images with signal suppression paralleling normal adipose tissue. Interdigitations with skeletal muscle are pathognomonic for intramuscular lipomas and can also be seen on MRI. Additionally, lipomas are distinguishable from well-differentiated liposarcomas by radiographic features such as the presence of thick septa, the presence of nodular or non-adipose areas, and decreased adipose composition (<75%) [14]. However, MRI is not sufficient to distinguish between well-circumscribed and infiltrative forms of intramuscular lipomas as they do not completely correspond to histological findings [15].

Histologically, intramuscular lipomas can be divided into three subtypes: infiltrative, well-defined, or mixed type [2]. Infiltrative intramuscular lipomas show mature uniform adipocytes with irregular infiltrations between striated muscles, oftentimes replacing the muscle [2]. In contrast, well-defined intramuscular lipomas are discrete masses of mature adipocytes with clear demarcations from muscle [2]. No entrapment of muscle or infiltration is present and fibrous stroma condenses to form a capsule, and in both types, there is no nuclear atypia or lipoblasts [2]. The mixed type contains regions of both infiltrative margins and well-demarcated encapsulated areas [2].

Small, stable, asymptomatic lipomas may be safely observed. For large, changing, or symptomatic intramuscular lipomas, surgical excision is the preferred treatment. During surgery, marginal excision of well-demarcated areas and wide excision with a margin of infiltrative regions is recommended. Reported recurrence of intramuscular lipomas after surgical excision is variable with recurrence rates of 3-62.5% [16]. Studies with a high recurrence rate were performed in an era where lipoblasts were required for the diagnosis of atypical lipomatous tumors, leading to the misclassification of well-differentiated liposarcomas as intramuscular lipomas. However, it is still essential to be prudent in wide excision of margins, particularly in areas of infiltration seen in preoperative imaging as infiltrative subtypes lend to increased recurrence rates, and in clinical follow-ups [17].

## Conclusions

Intramuscular lipomas are a rare subtype of lipomas with an incidence of less than 1% of all lipomas. Of these lipomas, the majority occur between the ages of 40 and 70 years. Since 2002, only eight intramuscular lipomas have been reported in the pediatric population on PubMed, and only three can be described as giant. Effective treatment of giant intramuscular lipomas utilize advanced imaging with wide surgical excision. This patient represents a rare case of a large intramuscular lipoma and one of the first described in a teenager.
